# Preventive CTLA-4-Ig Treatment Reduces Hepatic Egg Load and Hepatic Fibrosis in *Schistosoma mansoni*-Infected Mice

**DOI:** 10.1155/2019/1704238

**Published:** 2019-12-16

**Authors:** Martina Sombetzki, Anne Rabes, Miriam Bischofsberger, Franziska Winkelmann, Nicole Koslowski, Cindy Schulz, Emil C. Reisinger

**Affiliations:** Department of Tropical Medicine, Infectious Diseases and Nephrology, University Medical Center Rostock, Rostock, Germany

## Abstract

**Background:**

Hepatic fibrosis and granuloma formation as a consequence of tissue entrapped eggs produced by female schistosomes characterize the pathology of *Schistosoma mansoni* infection. We have previously shown that single-sex infection with female schistosomes mitigates hepatic fibrosis after secondary infection. This was associated with an increased expression of cytotoxic T-lymphocyte-associated protein-4 (CTLA-4), known as a negative regulator of T cell activation. Based on these findings, we hypothesized that administration of agonistic CTLA-4-Ig (Belatacept) is capable to prevent and/or treat hepatic fibrosis during schistosomiasis.

**Methods:**

Mice were infected with 50 *S. mansoni* cercariae and CTLA-4-Ig, or appropriated control-Ig was administered for 4 weeks. Preventive treatment started 4 weeks after infection, before onset of egg production, and therapeutic treatment started 8 weeks after infection when hepatic fibrosis was already established.

**Results:**

When given early after infection, livers of CTLA-4-Ig-treated mice showed significantly reduced collagen deposition and decreased expression of profibrotic genes in comparison to controls. In addition, administration of CTLA-4-Ig suppressed the inflammatory T cell response in infected mice. If therapy was started at a later time point when fibrogenesis was initiated, CTLA-4-Ig had no impact on hepatic fibrosis.

**Conclusion:**

We could demonstrate that an early preventive administration of CTLA-4-Ig suppresses effector T cell function and therefore ameliorates liver fibrosis. CTLA-4-Ig administration after onset of egg production fails to treat hepatic fibrosis.

## 1. Introduction

Schistosomiasis is a debilitating tropical disease caused by infection with trematode worms of the genus *Schistosoma* spp.. Currently, more than 200 million people, mostly in the tropic and subtropics, are affected; more than 700 million people in 78 countries are at risk of the infection [1]. The larvae of *Schistosoma (S.) mansoni*, one of the most common *Schistosoma* species besides *S. haematobium*, penetrate the skin of their hosts and migrate via the blood circulation, transiting the lungs to reside as adults in the mesenteric veins, where they mate and lay eggs around 5 weeks after infection. Parts of these eggs enter the portal circulation and are entrapped within the small liver sinusoids. Here, they induce a perioval granulomatous reaction resulting in severe hepatic fibrosis characterized by the excessive deposition of extracellular matrix proteins [2].

*S. mansoni*-induced hepatic fibrosis is of high relevance among chronic liver diseases worldwide. The pathology is mainly induced by cellular immune responses to tissue-entrapped eggs and orchestrated by CD4^+^ T cells. The early immune reaction to adult worm antigens is dominated by proinflammatory Th1 cytokines, but shifts towards a Th2-biased immune response following egg deposition. The granulomatous reaction is characterized by the infiltration of CD4^+^ Th2 cells, eosinophils, and alternatively activated macrophages, as well as the production of Th2 cytokines (IL-4, IL-5, and IL-13) promoting tissue fibrosis [3, 4]. During subsequent chronic infection, granuloma formation is downregulated by an upcoming regulatory T cell response, while sustained Th2-driven fibrotic response to egg antigens leads to clinical anomalies such as portal hypertension and subsequent ascites and life-threatening esophagus varices ruptions [5, 6].

The cytotoxic T-lymphocyte-associated antigen-4 (CTLA-4) is known as a crucial negative regulator of T-cell activation and proliferation. It acts as an antagonist of CD28-ligand interactions by competitive binding to CD80 and CD86 on antigen-presenting cells [7]. The ability of CTLA-4 to selectively suppress T-cell-mediated immune responses has made it a promising target for therapeutic interventions. Antagonistic CTLA-4 antibodies, such as ipilimumab, increase immune activation and are successfully used in tumor therapy [8, 9], whereas agonistic CTLA-4 fusion proteins, like commercially available belatacept and abatacept, act immunosuppressive. Treatment with belatacept has been shown to prevent rejection of allografts, particularly renal transplants [10, 11], and abatacept is used to treat rheumatoid arthritis [12, 13].

With respect to murine *S. mansoni* infections, blocking of CTLA-4 during acute infection was associated with significant weight loss and altered type 2 cytokine responses indicating the crucial importance of this regulator during *S. mansoni* infection [14]. Moreover, we recently reported that single-sex infection with female *S. mansoni* cercariae mitigates hepatic fibrosis after secondary infection, which was associated with an increased expression of CTLA-4 in these mice [15, 16].

We therefore hypothesized that a primary infection with female *S. mansoni* and the related antifibrotic effect can be mimicked by a CTLA-4-Ig administration. We performed two experimental approaches: (i) preventive CTLA-4-Ig treatment, starting at week 4 after infection and (ii) therapeutic CTLA-4-Ig treatment starting at week 8 after infection to investigate the therapeutic potency of CTLA-4-Ig in counteracting the profibrotic immune reactions. We herein demonstrated that preventive, but not therapeutic, CTLA-4-Ig treatment ameliorated hepatic fibrosis.

## 2. Methods

### 2.1. Ethics Statement

All animal experiments were performed in strict accordance with the German regulations of the Society for Laboratory Animal Science and the European Health Law of the Federation of Laboratory Animal Science Associations. The protocol was approved by the local committee on animal care and use (7221.3-1-034/18-1). All efforts were made to minimize the suffering of animals.

### 2.2. Mice Infection and Study Design

**Figure 1:**
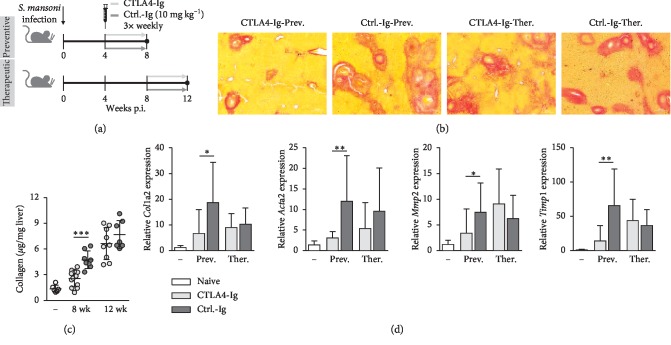
Preventive CTLA-4-Ig treatment reduces hepatic fibrosis, but has no therapeutic effect. (a) Schematic of *S. mansoni*-infected mice receiving either CTLA-4-Ig or ctrl.-Ig. (10 mg/kg) for 4 weeks 3 times weekly (preventive and therapeutic approach). (b) Representative images of liver sections stained with Sirus-Red are shown; 100x magnification. (c) Collagen deposition in livers of mice was quantified by measurement of hydroxyproline (*n* = 6–12 out of 4 independent experiments). (d) Relative gene expression of *Col1a2, Acta2, Mmp2,* and *Timp1* in livers of mice was determined by real-time PCR (*n* = 6–9, performed in triplicates). Data are represented as mean ± SD from triplicate data.

Eight-week-old female C57BL/6 mice were percutaneously infected with 50 cercariae of *S. mansoni* (Belo Horizonte strain) obtained from our in-house cycle of infected *Biomphalaria glabrata* snails (Brazilian strain) as previously described [15]. For treatment, belatacept (Nulojix, Bristol-Myers Squibb, Germany) and appropriate control antibodies (MP Biomedicals/Fisher scientific, Germany) were diluted in PBS (100 *μ*g/ml). Mice were administered 10 mg/kg belatacept or control-Ig three times a week by intraperitoneal injection for four weeks [16]. Preventive treatment started 4 weeks after infection (p.i.), and mice were sacrificed 8 weeks p.i.. Therapeutic treatment was started after egg deposition 8 weeks p.i., and mice were sacrificed 12 weeks p.i. (Figure 1(a)). We determined the following group sizes: uninfected naïve mice, *n* = 6; *S. mansoni*-infected mice, *n* = 12. Since not all animals in the therapeutically treated groups (scarification 12 weeks p.i.) were successfully infected (no granuloma formation and no increase of spleen and liver), we excluded these mice from the analysis. Due to the step-by-step experimental procedure, the results of this study were reproduced several times. On each processing day, we analyzed at least 2 to 3 animals of each group to ensure comparability.

### 2.3. Assessment of Pathology

The total amount of collagen in weighted liver fractions was quantified based on the colorimetric detection of hydroxyproline using a Quickzyme Total Collagen assay kit (Quickzyme Biosciences) according to the manufacturer's instructions. For histological evaluation, a standardized part of the right liver lobe was fixed in 10% neutral buffered formalin solution (Sigma-Aldrich) and embedded in paraffin. Thin sections of 5 *μ*m were stained for collagen with Sirius red (SR) or with haematoxylin/eosin (H&E). Granuloma size was determined using ImageJ software (v1.47v; National Institutes of Health, USA).

The relative weight of spleens and livers was expressed as the ratio of organ to body weight. Serum biochemistry for alanine aminotransferase (ALT), aspartate aminotransferase (AST), and alkaline phosphatase (AP) was performed using a UniCel® DxC 800 Synchron® Clinical System (Beckman Coulter GmbH). Total egg numbers were assessed by microscopic evaluation (100-fold magnification) of weighted liver fractions.

### 2.4. Quantitative RT-PCR

Total RNA was isolated from liver tissue (RNeasy Plus Mini Kit, Qiagen) and reversely transcribed into cDNA using a High-capacity cDNA Reverse Transcriptase Kit (Thermo Fisher) according to the manufacturer's instructions. Real-time PCR (RT-PCR) was performed using the following TaqMan Gene Expression Assays: *Col1a2* Mm00483888; *Acta2* Mm00725412; *Mmp2* Mm00439498; *Timp1* Mm01341361; *Il13* Mm00434204; *Il4* Mm00445259; *Infg* Mm01168134; and *Il10* Mm01288386 (Thermo Fisher). Gene expression values were normalized to the endogenous reference gene *Gapdh* (Rodent GAPDH control reagent, ThermoFisher) and presented as normalized, relative expression values to naive controls.

### 2.5. Cell Preparation

Single-cell suspensions were prepared by passing the spleen through a cell strainer (100 *μ*m) followed by PBS washing and erythrocytes lysis with RBC lysis buffer (BioLegend). Cells were washed twice with PBS and cell numbers were quantified using a CASY TT cell counter (OLS-Omni Life Science).

### 2.6. Flow Cytometry

Cells were stained with a Zombie Red™ Fixable Viability Kit (BioLegend) for 15 min at RT in PBS followed by incubation with appropriate fluorochrome-conjugated antibodies for 20 min at 4°C in FACS buffer (PBS + 3% FCS). The following antibodies were used: lymphoid panel anti-CD3-APC (clone 145-2C11), anti-CD4-PerCP-Cy5.5 (clone RM4-4), anti-CD8-PE-Cy7 (clone 53-6.7), anti-CTLA-4-PE (clone UC10-4B9), IgG Isotope Ctrl.-PE (clone HTK888); myeloid panel anti-CD11b-APC (clone M1/70), anti-CD11c-Alexa488 (clone N418), anti-F4/80-PE-Cy7 (clone BM8), and anti-CD86-BV605 (clone GL-1). All antibodies were purchased from BioLegend. After washing, flow cytometric analysis was performed using FACS Aria™ IIIu (BD Bioscience), and data were analyzed by FlowJo software (v10.0.7, Tree Star Inc., CA, USA). Live cells were differentiated by gating on the following cell populations: T cells (CD3^+^/CD4^+^ or CD3^+^/CD8^+^), macrophages (CD11b^+^F4/80+), and dendritic cells (CD11c^+^). A representative gating strategy for CTLA-4 is given in supplementary [Supplementary-material supplementary-material-1].

### 2.7. Cytokine ELISAs

For assessment of cytokine production, isolated splenocytes were cultured in RPMI 1640 medium supplemented with 10% FCS, 25 mM HEPES and antibiotics. Cells were stimulated by an in-house generated 10 *μ*g/ml *S. mansoni* soluble worm antigen prepartion (SWAP) for 72 h at 37°C [16]. Cytokines in cell-free supernatants were quantified using DuoSet ELISAs Kits (R&D Systems) detecting IL-13, IL-4, INF-*γ*, or IL-10 according to the manufacturer's protocol.

### 2.8. Statistics

Statistical analysis was performed using GraphPad Prism 5.0 (GraphPad Software, La Jolla, CA). Values are expressed as mean + SD. Differences between groups were analyzed by Mann–Whitney *U* test, and the *p* values < 0.05 were considered statistically significant. ^*∗*^*p* < 0.05, ^*∗∗*^*p* < 0.01, and ^*∗∗∗*^*p* < 0.001.

## 3. Results

### 3.1. Preventive CTLA-4-Ig Treatment Reduces Hepatic Fibrosis but Has No Therapeutic Effect

To investigate whether CTLA-4 impacts the development of hepatic fibrosis during Schistosomiasis, we treated *S. mansoni*-infected mice with CTLA-4-Ig or appropriate control antibodies (ctrl.-Ig) for 4 weeks starting at 4 weeks p.i. (preventive approach) or 8 weeks p.i. (therapeutic approach) (Figure 1(a)). Histological evaluation of Sirus-Red (SR) stained liver slices revealed that preventive administration of CTLA-4-Ig resulted in a reduction of SR-positive areas and portoportal bridging compared to the control group. However, there were no pronounced differences between the CTLA-4-Ig and ctrl.-Ig treated groups when therapeutic treatment was performed (Figure 1(b)). To underpin these observations, hepatic fibrosis was quantified by the measurement of hydroxyproline as a marker for collagen deposition. Hydroxyproline levels were significantly diminished in mice preventively, but not therapeutically, treated with CTLA-4-Ig compared to controls. As expected, collagen deposition increased over time and was the highest in livers of mice at 12 weeks p.i. (therapeutic approach) (Figure 1(c)). In line with these findings, expression levels of fibrosis-associated genes *collagen type I alpha 2 (Col1a2,) alpha-actin-2 (Acta2), matrix metalloproteinase-2 (Mmp2),* and *tissue inhibitor of metalloproteinases (Timp1)* were significantly decreased in livers of mice after preventive, but not therapeutic, CTLA-4-Ig treatment in comparison to controls (Figure 1(d)). Overall, these data demonstrate that preventive CTLA-4-Ig administration efficiently ameliorates hepatic fibrosis of *S. mansoni*-infected mice.

### 3.2. Hepatosplenomegaly and Egg Load Are Decreased in Mice Preventively Treated with CTLA-4-Ig

**Figure 2:**
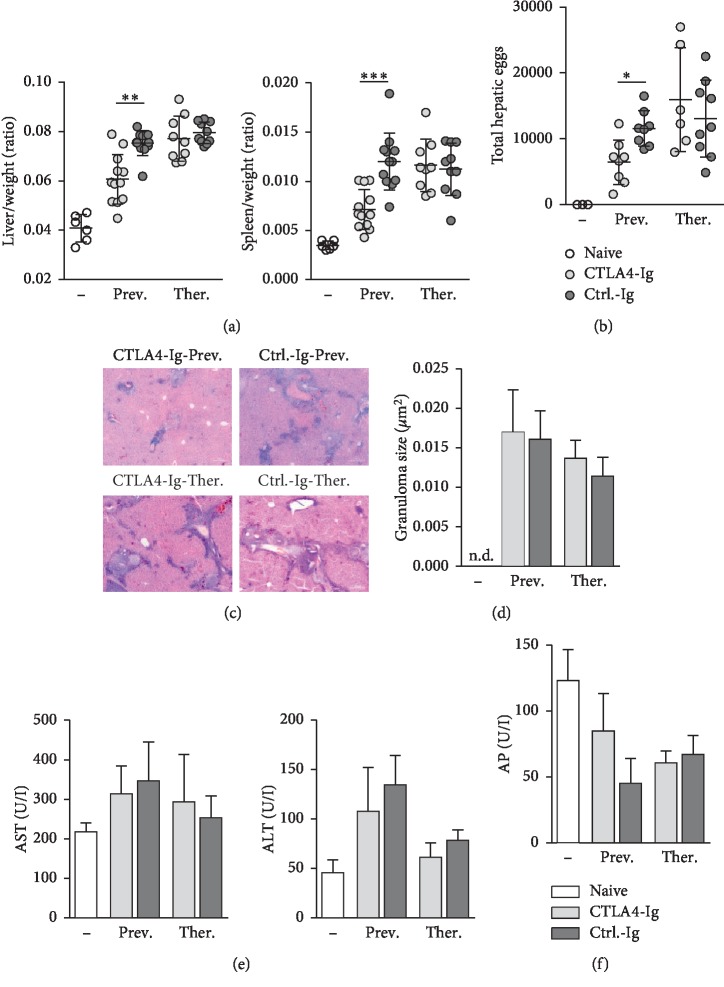
Hepatosplenomegaly and egg load are decreased in mice preventively treated with CTLA-4-Ig. (a) Relative organ size of livers and spleens was expressed as a ratio to body weight (*n* = 6–12 out of 4 independent experiments). (b) Numbers of total hepatic eggs were counted (*n* = 6–9 out of 4 independent experiments). (c) Representative images of liver sections stained with haematoxylin/eosin are shown; 100x magnification. (d) Size of hepatic granulomas were quantified by using Image*J* software (*n* = 5–7 out of 4 independent experiments). (e) Serum levels of aspartate aminotransferase (AST), alanine aminotransferase (ALT), and (f) alkaline phosphatase (AP) were determined (*n* = 4–6 out of 4 independent experiments). Data are represented as mean ± SD from duplicate data.

Since preventive CTLA-4-Ig treatment was capable to reduce hepatic fibrosis during schistosomiasis, we further analyzed the impact of the treatment on disease progression. Hepatosplenomegaly, characterized by a simultaneous enlargement of the liver and the spleen, was significantly less pronounced in mice preventively, but not therapeutically, treated with CTLA-4-Ig compared to controls (Figure 2(a)). In addition, hepatic egg load was significantly reduced in these mice (Figure 2(b)). Infection with *S. mansoni* resulted in a uniform appearance of egg granulomas in the livers of infected mice (Figure 2(c)). Perioval granulomas displayed comparable sizes in all experimental groups (Figure 2(d)). Liver transaminases (AST and ALT), as a marker for hepatocellular damage, were slightly elevated in the serum of infected mice compared to naïve mice. However, all values were below clinical relevance and not significantly different. Serum levels of AP were not affected by the infection (Figure 2(d)). These results indicate that preventive CTLA-4-Ig treatment improves the clinical picture of schistosomiasis.

### 3.3. CTLA-4-Ig Treatment Leads to a Reduction in Total Cell Counts and CD4^+^ T Cells in the Spleens of Mice

**Figure 3:**
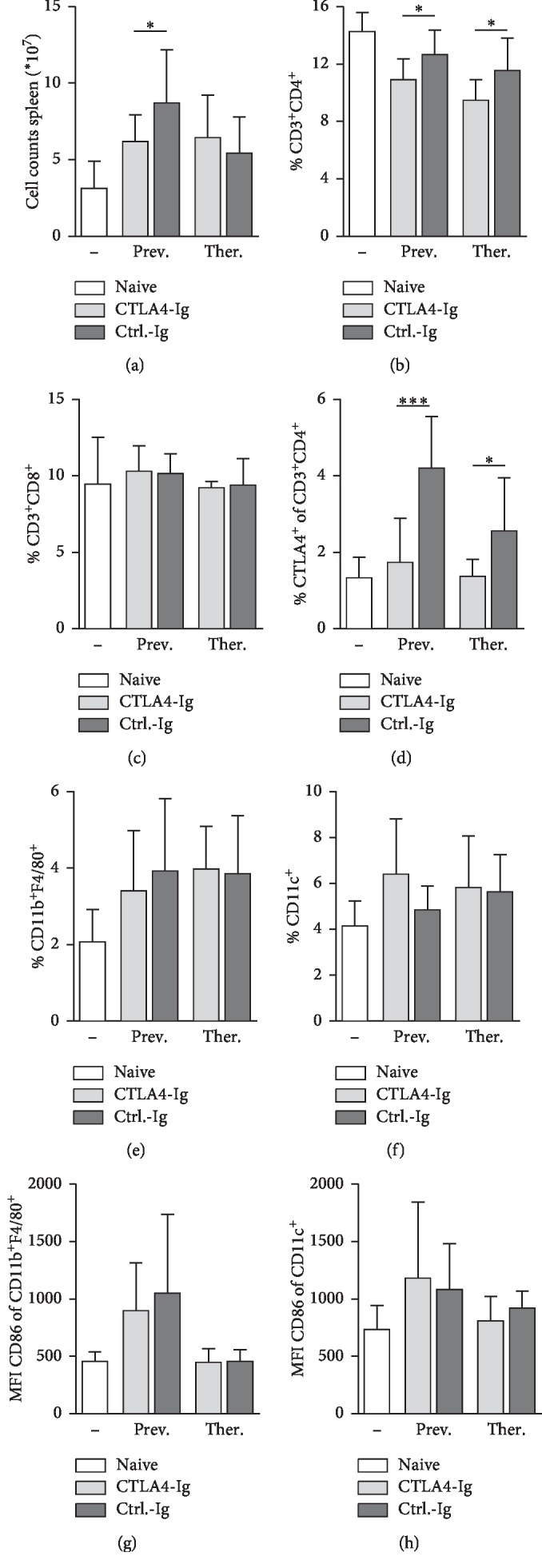
CTLA-4-Ig treatment leads to a reduction in total cell counts and CD4^+^ T cells in the spleens of mice. (a) Numbers of spleen cells were quantified using a CASY TT cell counter and ((b)-(h)) analyzed by flow cytometry (*n* = 6–12 out of 4 independent experiments). The percentage of (b) CD4^+^ T cells, (c) CD8^+^ T cells, (d) CTLA-4^+^ CD4^+^ T cells, (e) macrophages (M*ϕ*s), and (f) dendritic cells (DCs) of viable cells was depicted. The mean fluorescence intensity (MFI) of CD86 expression on M*ϕ*s and DCs was quantified. Data are represented as mean + SD from duplicate data.

To characterize the role of immunosuppressive CTLA-4 on inflammatory immune cell recruitment, we analyzed the spleens of mice by flow cytometry. Preventive administration of CTLA-4-Ig led to a significant reduction of total cell numbers in the spleens compared to controls. However, administration of CTLA-4-Ig had no impact on cell numbers when the treatment was started after egg deposition (therapeutic approach) (Figure 3(a)). Treatment with CTLA-4-Ig selectively decreased the percentage of CD4^+^ T cells, but not CD8^+^ T cells, in *S. mansoni*-infected mice (Figures 3(b) and 3(c)). Moreover, we observed that mice receiving ctrl.-Ig upregulate the expression of CTLA-4 on the surface of CD4^+^ T cells during infection in contrast to CTLA-4-Ig-treated mice (Figure 3(d)). With regard to myeloid cells, CTLA-4-Ig administration neither affected the percentage of antigen-presenting F4/80^+^ macrophages and CD11^+^ dendritic cells within spleens nor the expression of the costimulatory molecule and CTLA-4-ligand CD86 on these cells, which indicates that the functionality of macrophages and dendritic cells is not affected by the treatment (Figure 3(e)–3(h)). Taken together, these observations prove that the administration of CTLA-4-Ig efficiently suppresses the CD4^+^ T cell response *in vivo.*

### 3.4. Preventive CTLA-4-Ig Treatment Impairs Cytokine Production by Splenocytes and Gene Expression of Cytokines in the Livers

**Figure 4:**
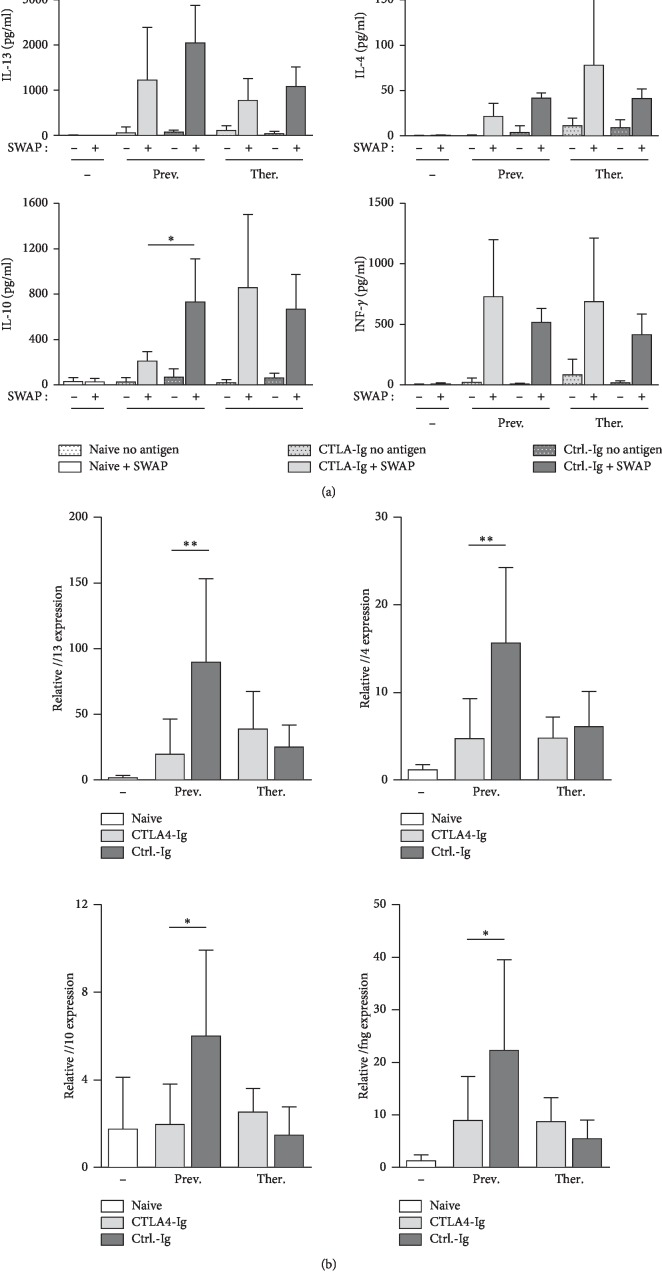
Cytokine production by splenocytes and expression of cytokines in the livers of mice preventively treated with CTLA-4-Ig are impaired. (a) Splenocytes were isolated and stimulated with 10 *μ*g/ml SWAP (soluble worm antigen preparation). Supernatants were collected after 72 h and amounts of IL-13, IL-4, INF-*γ,* and IL-10 (*n* = 4–9 out of 3 independent experiments) were quantified by ELISA (*n* = 4–9). (b) Relative gene expression of *Il13*, *Il4*, *Ifng,* and *Il10* in livers of mice was determined by real-time PCR (*n* = 6–9). Data are represented as mean + SD from duplicate data.

Considering the fact that CTLA-4-Ig suppresses the cellular immune response in *S. mansoni*-infected mice, we next examined whether the treatment influences the cytokine production of isolated splenocytes and the expression of cytokines in the liver. The secretion of Th2 key cytokine IL-13, known to promote fibrogenesis, as well as regulatory IL-10, was decreased in the supernatants of SWAP-stimulated splenocytes of preventively CTLA-4-Ig-treated mice in comparison to ctrl.-Ig-treated control group, whereas INF-*γ* secretion was not affected by the treatment (Figure 4(a)). In addition, expression levels of *Il13, Il4, Infg,* and *Il10* in the livers of these mice were significantly downregulated (Figure 4(b)). The cytokine response of mice in the therapeutically treated groups was diminished in comparison to the preventive ctrl.-Ig group. However, cytokine levels were not affected by the therapeutical CTLA-4-Ig treatment (Figure 4(a) and 4(b)). These data show that the production of profibrotic cytokines is impaired in mice preventively treated with CTLA-4-Ig.

## 4. Discussion

The current study was performed as a proof-of-principle study. We previously demonstrated that the antifibrotic effect of single-sex infection with female schistosomes is associated with increased CTLA-4 expression in livers of these mice [15]. Based on these data, it was obvious for us to investigate the potential direct antifibrotic effect of CTLA-4 on *S. mansoni*-induced hepatic fibrosis by mimicking the effect of female worms. Herein, we demonstrated that an early preventive treatment with CTLA-4-Ig (belatacept) in mice leads to (i) reduction of hepatic fibrosis, (ii) diminished disease progression, (iii) reduced hepatic egg load, and (iv) an impaired immune response characterized by decreased immune cell recruitment and cytokine production. However, CTLA-4-Ig administration was not capable to treat ongoing hepatic fibrosis.

In mansonian schistosomiasis, hepatic fibrosis is initiated by vigorous granulomatous responses to tissue-entrapped parasite eggs that is mainly orchestrated by cross-regulatory CD4^+^ T cell. Although an appropriate Th1 response to *S. mansoni* larvae is associated with high protection levels [17] and Th2 response to soluble egg antigens is known to promote excessive fibrotic organ damage [18], there are actually no good or bad T cell responses to *Schistosoma* larvae or eggs. Earlier studies in experimental schistosomiasis have shown the essential role for CD4^+^ T cells in granuloma formation and disease [19]. Excessive polarization to either Th1 or Th2 by knocking down certain type 2 cytokines was shown to impede granulomatous response leading to 100% mortality [20–22]. The picture of formally announced Th1/Th2 paradigm is obsolete due to the discovery of a more complex pattern of regulation and interplay of different CD4^+^ T cell subsets [23]. It has been shown that immunotherapy by administration of specific antibodies or agonists have remarkable effects on the establishment of hepatic fibrosis following *S. mansoni* infection. Substitution of Th2 response by Th1, induced by IL-12 administration, is able to prevent excessive tissue fibrosis [24]. In addition, direct interference with the type 2 cytokine IL-13 by IL-13 inhibitor sIL-13Rα2-Fc leads to significant reduction of hepatic fibrosis in a mouse model of *S. mansoni* infection [25].

One important regulator of T cell function is the CTLA-4 contributing to the suppressor function of regulatory and conventional CD4^+^ T cells [26]. CTLA-4 is expressed on the T cell surface and binds to the B7 molecule on antigen-presenting cells leading to an inhibitory signal to the activated T cell by limiting the production of the T cell growth factor IL-2 [27]. CTLA-4^−/−^ mice developed a fatal disease characterized by massive proliferation of lymphocytes [28]. Vice versa, the administration of CTLA-4-Ig (belatacept or abatacept) exerts beneficial effects on a range of autoimmune disorders such as airway inflammation [29, 30], rheumatoid arthritis [31], and dermal fibrosis [32] or to prevent rejection of allografts, particularly renal transplants [33].

In its proper use (prevention of kidney transplant rejection), immunosuppressant Nulojix® (belatacept, CTLA-4) is a very expensive therapy. In consideration to *S. mansoni* infection in mice, it has been shown that blocking of CTLA-4 by anti-CTLA-4 antibodies leads to significant weight loss and altered Th2 response in these mice when administered during the acute stage of infection [14]. Using a single-sex infection model of *S. mansoni*, we have recently shown that an initial infection with female *S. mansoni* reduces hepatic fibrosis of a bisexual challenge infection [15]. We concluded that this inhibited Th2 response and the antifibrotic effect was due to a significant increase of CTLA-4 expression in the livers of these mice. To our knowledge, there is only one study in mice showing similar results in regard to impairment of Th2 responses due to CTLA-4-Ig injection in experimental *Nippostrongylus brasiliensis* infection [34]. In humans, it has been shown that CTLA-4 is upregulated following *Schistosoma* spp. infection, which was associated with a reduced incidence of Th2-driven allergic diseases [35, 36].

The reduction of hepatic fibrosis, observed in this study, must be viewed from different angles. On one hand side, we have shown a significant reduction of the hepatic egg load in the prevention group. This alone can be the cause of the reduction of hepatic fibrosis. The reason for the reduced egg load might be related to a disturbed maturation of the worms due to the immunosuppressive effect of CTLA-4. Harrison and Doenhoff have shown that a disturbed CD4 T cell response by the administration of immunosuppressive substances negatively affects the fecundity of female worms, which in turn leads to reduced egg production [37]. On the other hand side, a selective costimulatory blockage of CD28-mediated activation of T cells might exert a direct antifibrotic effect. The spreading of fibrosis mainly depends on Th2 expansion. Therefore, fibrogenesis, as a fibroproliferating process, might be directly affected. The granulomas consist of different cell types that are not influenced by the specific effect of costimulatory blockage of CD28-mediated T cell activation. This could explain the unchanged granuloma sizes in our experimental groups. However, if the egg numbers are correlated to the amount of hydroxyproline, the differences in hydroxyproline levels were fading. This fact points rather to the reduced egg load as the main cause for the reduced fibrosis. Another point that could play a role in the development of hepatic fibrosis is the fact that in this study the right hepatic lobe was used to quantify hydroxyproline levels, although in human infections the left hepatic lobe is more conspicuous. However, this study focused on the analysis of relative changes in the degree of fibrosis and less with absolute changes. Several studies indicate on the interaction of regulatory T cells and matrix metalloproteinases (MMP) with emphasis on tumorigenesis. Anti-CTLA-4 treatment, not at least due to the removal/reduction of immune tolerance, is a very promising anticancer therapy [38]. Increased collagen synthesis along with a downregulation of proteolytic enzymes, especially matrix metalloproteases (MMP), and upregulation of tissue inhibitors of MMP (TIMP) may contribute to hepatic fibrosis [39–41]. During *S. mansoni* infection, MMP-2 and TIMP-1 expression levels are generally upregulated, where the expressions levels for TIMP-1 are usually higher than those for MMP-2, as evidence of an ongoing fibrotic process. Interestingly, we could observe inverted proportions in both, the preventive and the therapeutic group, indicating fibrolytic processes. Since CD4^+^ T cells itself produce MMPs [38], a reduction of CD4^+^ cells in our setting might explain the observed reduction in MMP-2. However, the reduction of hepatic fibrosis was seen in the preventive group, exclusively. In the “therapeutic” group, we could only see a trend reduction of fibrosis in all measured parameters.

In the current study, we could show that there is an overall reduction of CD4^+^ T cells within spleens of infected mice that received CTLA-4-Ig during acute infection. These CD4^+^ T cells express less CTLA-4 compared to ctrl.-Ig groups, indicating that the treatment was effectively working *in vivo*. In addition, *ex vivo* analysis of SWAP-stimulated splenocytes displayed a reduced Th2 cytokine production, as an important trigger of fibrosis. The use of SWAP instead of SEA in this study is due to experiments prior to the presented study which showed that SWAP elicits the most reproducible results compared to SEA or T-cell-receptor-specific (CD3/CD28) or nonspecific (PMA/ionomycin) stimulation methods. This may be explained by the fact that the protocol used for SWAP production [42] involves the processing of worm pairs, and therefore parasite egg antigens are most likely present.

The reduced inflammation observed in livers and spleens due to immunosuppressive CTLA-4 administration led to reduction of regulatory IL-10, which is crucial for emergence of Treg responses during *Schistosoma* spp. infection [43, 44]. It has been show that reduced IL-10 levels are associated with increased resistance to reinfection [45, 46]. However, the observed low IL-10 values, artificially induced by belatacept in this study, might have the opposite effect as well and induce a higher vulnerability to the infection in human infections. IL-10 is undoubtedly a major molecule involved in immunosuppression and development of a modified Th2 response [43]. In this study, the development of an inflammatory response against the parasites and eggs has been severely disarranged in the prevention group. We suggest that a certain level of inflammation or a sequence of inflammatory processes during the infection is needed to initiate the regulatory potential of IL-10. In a system of artificially induced immunosuppression, regulation by IL-10 might not be initiated or failed. We therefore assume that an artificial immunosuppression by belatacept will disturb the balance between Th1/Th2/Treg considerably seen by an antifibrotic effect in the prevention group but no effect in the therapeutic group.

The question remains whether the effect of belatacept on CD4^+^ T cell response in regard to the reduction of hepatic fibrosis is restorable in case of a CTLA-4-Ig treatment stop. We designed a follow-up study to analyze a potential long-term effect of belatacept (follow-up: CTLA-4-Ig, *n* = 6 and ctrl.-Ig, *n* = 4). We had to abandon that study 12 weeks p.i. since the animals reached critical termination criteria in regard to weight loss and mobility. It has been shown that in the absence of CD4^+^ T cells the granulomatous response is limited leading to increased hepatocyte damage [19]. However, the surrogate marker for liver injury (ALT and AST) did not indicate severe organ damage. We assume that these mice, due to the early administration of CTLA-4, generate a severe systemic inflammation due to the inability to develop an appropriate CD4^+^ T cell response.

In conclusion, this study points clearly to the important role of CTLA-4 as an important regulator of *S. mansoni*-induced fibrosis by modulating T-cell responses. However, our data show that early preventive CTLA-4-Ig treatment ameliorates liver fibrosis, but was not sufficient to treat ongoing fibrotic processes. In endemic areas, *S. mansoni* infection is not a once-only event but is very likely to occur regularly. A therapeutic effect of belatacept would therefore be, although rather unlikely due to its expense, a promising approach, especially in severe disease progressions. The more effective preventive treatment will be difficult to implement, considering that young children in particular are affected by the infection. Therefore, this study does not provide a probable tool, which could be considered for therapeutic treatment in human natural infection, but contributes a puzzle piece to the effect of CTLA-4 in *S. mansoni*-associated fibrogenesis and its role in disease progression.

## Data Availability

All data (original data) used to support the findings of this study are an integral part of this manuscript.

## Abbreviations

ALT: Alanine aminotransferase
AP: Alkaline phosphatase
AST: Aspartate aminotransferase
CTLA-4: Cytotoxic T-lymphocyte-associated protein-4
*S. mansoni*: *Schistosoma mansoni*
Th1: T helper cell response type 1
Th2: T helper cell response type 2

## Supplementary Materials

Supplementary Materials Supplementary Figure 1: representative FACS gating strategy for CD4^+^/CTLA-4^+^ cells in spleen homogenates. FACS plots: (A) gating of CD4^+^ T cells and (B) extracellular staining of CTLA-4 and appropriate control-IG on CD3^+^CD4^+^ T cells.

## References

[1] Who_Fact_Sheet WHO (2018). Schistosomiasis fact sheet. http://www.who.int/news-room/fact-sheets/detail/schistosomiasis.

[2] McManus D. P., Dunne D. W., Sacko M., Utzinger J., Vennervald B. J., Zhou X. N. (2018). Schistosomiasis. *Nature Reviews Disease Primers*.

[3] Kamdem S. D., Moyou-Somo R., Brombacher F., Nono J. K. (2018). Host regulators of liver fibrosis during human schistosomiasis. *Frontiers in Immunology*.

[4] Kaviratne M., Hesse M., Leusink M. (2004). IL-13 activates a mechanism of tissue fibrosis that is completely TGF-*β* independent. *The Journal of Immunology*.

[5] Rutitzky L. I., Hernandez H. J., Stadecker M. J. (2001). Th1-polarizing immunization with egg antigens correlates with severe exacerbation of immunopathology and death in schistosome infection. *Proceedings of the National Academy of Sciences*.

[6] Elbaz T., Esmat G. (2013). Hepatic and intestinal schistosomiasis: review. *Journal of Advanced Research*.

[7] Slavik J. M., Hutchcroft J. E., Bierer B. E. (1999). CD28/CTLA-4 and CD80/CD86 families. *Immunologic Research*.

[8] Force J., Leal J. H. S., McArthur H. L. (2019). Checkpoint blockade strategies in the treatment of breast cancer: where we are and where we are heading. *Current Treatment Options in Oncology*.

[9] Wojas-Krawczyk K., Kalinka E., Grenda A., Krawczyk P., Milanowski J. (2019). Beyond PD-L1 markers for lung cancer immunotherapy. *International Journal of Molecular Sciences*.

[10] Mulvihill M. S., Samy K. P., Gao Q. A. (2019). Secondary lymphoid tissue and costimulation-blockade resistant rejection: a nonhuman primate renal transplant study. *American Journal of Transplantation*.

[11] Sparkes T., Ravichandran B., Opara O. (2019). Alemtuzumab induction and belatacept maintenance in marginal pathology renal allografts. *Clinical Transplantation*.

[12] Otani K., Kurosaka D. (2019). Abatacept suppresses the telomerase activity of lymphocytes in patients with rheumatoid arthritis. *International Journal of Rheumatic Diseases*.

[13] Zou Q. F., Li L., Han Q. R., Wang Y. J., Wang X. B. (2019). Abatacept alleviates rheumatoid arthritis development by inhibiting migration of fibroblast-like synoviocytes via MAPK pathway. *European Review for Medical and Pharmacological Sciences*.

[14] Walsh C. M., Smith P., Fallon P. G. (2007). Role for CTLA-4 but not CD25^+^ T cells during Schistosoma mansoni infection of mice. *Parasite Immunology*.

[15] Koslowski N., Sombetzki M., Loebermann M. (2017). Single-sex infection with female *Schistosoma mansoni* cercariae mitigates hepatic fibrosis after secondary infection. *PLoS Neglected Tropical Diseases*.

[16] Ville S., Poirier N., Branchereau J. (2016). Anti-CD28 antibody and belatacept exert differential effects on mechanisms of renal allograft rejection. *Journal of the American Society of Nephrology*.

[17] Mountford A. P., Shires V. L., Anderson S. (1998). Interleukin-12 and protective immunity to schistosomes. *Brazilian Journal of Medical and Biological Research*.

[18] Fairfax K., Nascimento M., Huang S. C.-C., Everts B., Pearce E. J. (2012). Th2 responses in schistosomiasis. *Seminars in Immunopathology*.

[19] Hams E., Aviello G., Fallon P. G. (2013). The schistosoma granuloma: friend or foe?. *Frontiers in Immunology*.

[20] Wynn T., Cheever A. W. (1995). Cytokine regulation of granuloma formation in schistosomiasis. *Current Opinion in Immunology*.

[21] Hoffmann K. F., Cheever A. W., Wynn T. A. (2000). IL-10 and the dangers of immune polarization: excessive type 1 and type 2 cytokine responses induce distinct forms of lethal immunopathology in murine schistosomiasis. *The Journal of Immunology*.

[22] Brunet L. R., Finkelman F. D., Cheever A. W., Kopf M. A., Pearce E. J. (1997). IL-4 protects against TNF-alpha-mediated cachexia and death during acute schistosomiasis. *Journal of Immunology*.

[23] Bouchery T., Kyle R., Ronchese F., Le Gros G. (2014). The differentiation of CD4(+) T-helper cell subsets in the context of helminth parasite infection. *Frontiers in Immunology*.

[24] Wynn T. A., Cheever A. W., Jankovic D. (1995). An IL-12-based vaccination method for preventing fibrosis induced by schistosome infection. *Nature*.

[25] Chiaramonte M. G., Donaldson D. D., Cheever A. W., Wynn T. A. (1999). An IL-13 inhibitor blocks the development of hepatic fibrosis during a T-helper type 2-dominated inflammatory response. *Journal of Clinical Investigation*.

[26] Tai X., Van Laethem F., Pobezinsky L. (2012). Basis of CTLA-4 function in regulatory and conventional CD4^+^ T cells. *Blood*.

[27] Tsuyuki S., Tsuyuki J., Einsle K., Kopf M., Coyle A. J. (1997). Costimulation through B7-2 (CD86) is required for the induction of a lung mucosal T helper cell 2 (TH2) immune response and altered airway responsiveness. *The Journal of Experimental Medicine*.

[28] Mandelbrot D. A., McAdam A. J., Sharpe A. H. (1999). B7-1 or B7-2 is required to produce the lymphoproliferative phenotype in mice lacking cytotoxic T lymphocyte-associated antigen 4 (CTLA-4). *The Journal of Experimental Medicine*.

[29] Padrid P. A., Mathur M., Li X. (1998). CTLA4Ig inhibits airway eosinophilia and hyperresponsiveness by regulating the development of Th1/Th2 subsets in a murine model of asthma. *American Journal of Respiratory Cell and Molecular Biology*.

[30] Jiménez-Alvarez L., Arreola J. L., Ramírez-Martínez G. (2011). The effect of CTLA-4Ig, a CD28/B7 antagonist, on the lung inflammation and T cell subset profile during murine hypersensitivity pneumonitis. *Experimental and Molecular Pathology*.

[31] Jansen D. T., el Bannoudi H., Arens R. (2017). Abatacept decreases disease activity in a absence of CD4(+) T cells in a collagen-induced arthritis model. *Arthritis Research & Therapy*.

[32] Ponsoye M., Frantz C., Ruzehaji N. (2016). Treatment with abatacept prevents experimental dermal fibrosis and induces regression of established inflammation-driven fibrosis. *Annals of the Rheumatic Diseases*.

[33] Ford M. L., Adams A. B., Pearson T. C. (2014). Targeting co-stimulatory pathways: transplantation and autoimmunity. *Nature Reviews Nephrology*.

[34] Harris N. L., Peach R. J., Ronchese F. (1999). CTLA4-Ig inhibits optimal T helper 2 cell development but not protective immunity or memory response to Nippostrongylus brasiliensis. *European Journal of Immunology*.

[35] van den Biggelaar A. H., van Ree R., Rodrigues L. C. (2000). Decreased atopy in children infected with Schistosoma haematobium: a role for parasite-induced interleukin-10. *The Lancet*.

[36] Araujo M. I., Hoppe B., Medeiros M. (2004). Impaired T helper 2 response to aeroallergen in helminth-infected patients with asthma. *The Journal of Infectious Diseases*.

[37] Harrison R. A., Doenhoff M. J. (1983). Retarded development of Schistosoma mansoni in immunosuppressed mice. *Parasitology*.

[38] Benson H. L., Mobashery S., Chang M. (2011). Endogenous matrix metalloproteinases 2 and 9 regulate activation of CD4^+^ and CD8^+^ T cells. *American Journal of Respiratory Cell and Molecular Biology*.

[39] Loebermann M., Sombetzki M., Langner C. (2009). Imbalance of pro- and antifibrogenic genes and bile duct injury in murineSchistosoma mansoniinfection-induced liver fibrosis. *Tropical Medicine & International Health*.

[40] Schuppan D., Porov Y. (2002). Hepatic fibrosis: from bench to bedside. *Journal of Gastroenterology and Hepatology*.

[41] Hemmann S., Graf J., Roderfeld M., Roeb E. (2007). Expression of MMPs and TIMPs in liver fibrosis - a systematic review with special emphasis on anti-fibrotic strategies. *Journal of Hepatology*.

[42] Grenfell R. F. Q., Martins W. H., Silva-Moraes V. (2012). Antigens of worms and eggs showed a differentiated detection of specific IgG according to the time of Schistosoma mansoni infection in mice. *Revista da Sociedade Brasileira de Medicina Tropical*.

[43] Herbert D. B. R., Orekov T., Perkins C., Finkelman F. D. (2008). IL-10 and TGF-*β* redundantly protect against severe liver injury and mortality during acute schistosomiasis. *The Journal of Immunology*.

[44] Resende S. D., Magalhães F. C., Rodrigues-Oliveira J. L. (2019). Modulation of allergic reactivity in humans is dependent on *Schistosoma mansoni* parasite burden, low levels of IL-33 or TNF-*α* and high levels of IL-10 in serum. *Frontiers in Immunology*.

[45] Sadler C. H., Rutitzky L. I., Stadecker M. J., Wilson R. A. (2003). IL-10 is crucial for the transition from acute to chronic disease state during infection of mice with Schistosoma mansoni. *European Journal of Immunology*.

[46] Wilson M. S., Cheever A. W., White S. D., Thompson R. W., Wynn T. A. (2011). IL-10 blocks the development of resistance to Re-infection with schistosoma mansoni. *PLoS Pathogens*.

