# Supplementary material for: Preventive CTLA-4-Ig Treatment Reduces Hepatic Egg Load and Hepatic Fibrosis in Schistosoma mansoni-Infected Mice
Source: Biomed Res Int. 2019 Dec 16;2019:1704238. doi: 10.1155/2019/1704238 (PMC6948272; doi:10.1155/2019/1704238)
Supplement: Supplementary Materials — Supplementary Figure 1: representative FACS gating strategy for CD4+/CTLA-4+ cells in spleen homogenates. FACS plots: (A) gating of CD4+ T cells and (B) extracellular staining of CTLA-4 and appropriate control-IG on CD3+CD4+ T cells. [file 1704238.f1.pdf]

Supplementary Data Section

Title

"Preventive CTLA-4-Ig treatment reduces hepatic egg load and hepatic fibrosis in *Schistosoma mansoni* infected mice."

Authors

Martina Sombetzki, Anne Rabes, Miriam Bischofsberger, Franziska Winkelmann, Nicole Koslowski, Cindy Schulz, Emil C. Reisinger*.

Table of content

- **Supplementary Figure 1:** Representative FACS gating strategy for CD4^{+}/CTLA-4^{+} cells in spleen homogenates.

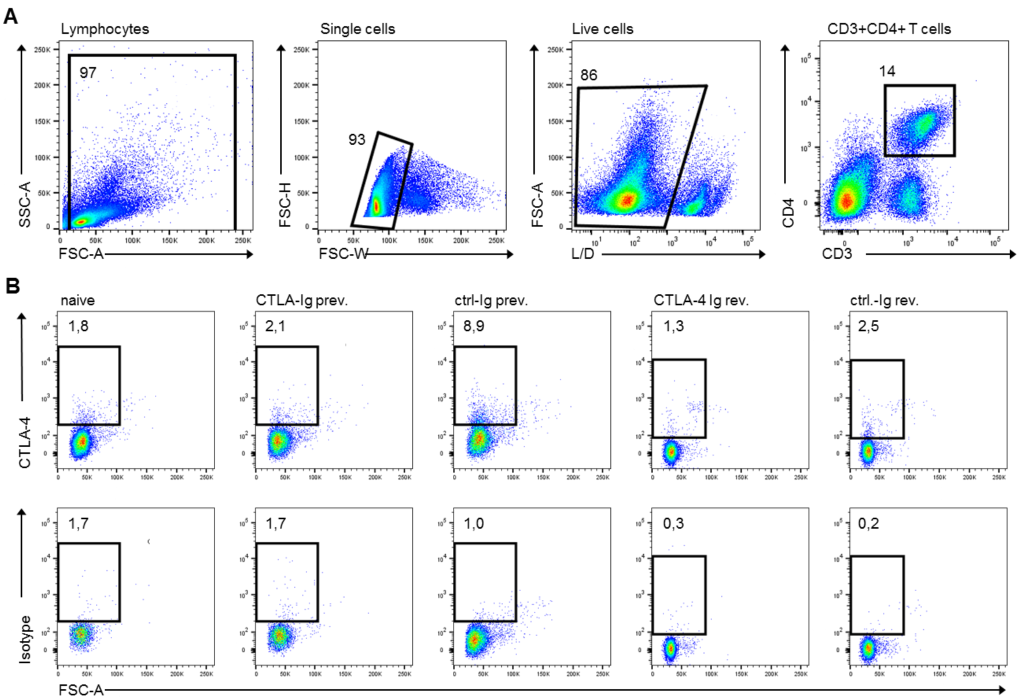

Supplementary Fig. 1: Representative FACS gating strategy for CD4^{+}/CTLA-4^{+} cells in spleen homogenates. FACS plots: (A) gating of CD4+ T cells and (B) extracellular staining of CTLA-4 and appropriate control-IG on CD3+CD4+ T cells.
